# Early-Onset Alzheimer's Disease Masquerading as Catatonia

**DOI:** 10.1155/2020/1493481

**Published:** 2020-09-12

**Authors:** Aljoharah Alakkas, Aaron Meyer, Eric Debbold, Raisa Yagudayeva, Jonathan Bui

**Affiliations:** University of California, San Diego, CA, USA

## Abstract

A 35-year-old woman with a history of sexual trauma was brought in by her family for further evaluation of depressive symptoms and progressive decline in activities of daily living. She was admitted to the inpatient psychiatric unit for the treatment of suspected catatonia. After failure to respond to standard medical treatment, she received an extensive workup, which ultimately revealed a PSEN1 mutation consistent with early-onset Alzheimer's disease. Diagnosis was challenging because of her young age, lack of reliable family history, and reports of recent sexual abuse by her biological father. This case is a cautionary reminder for clinicians that end stages of dementia can present similar to catatonia with mutism, lack of spontaneous movement, and refusal to eat. The clues to the diagnosis were profound cortical atrophy and lack of improvement with optimal medical management.

## 1. Case Report

A 35-year-old woman (pseudonym: LW) was brought to the emergency department on psychiatric hold for grave disability caused by functional decline in the context of reported sexual abuse.

LW arrived at the emergency department after an Adult Protective Services Welfare check was performed on her at home. Per her prehospital hold paperwork, “Received referral by APS due to client having mental health issues and unable to care for herself. On evaluation, client appeared disoriented, unable to state her DOB, current year, or day. Client had clothes worn backwards and is unable to dress by herself. If she is not fed by other, she will not eat or cook. Client will go to the bathroom on the floor.”

An emergency psychiatric consult was placed, and on interview, the patient was observed to be slowly ambulating in circles and exhibiting notable paucity in speech. When asked her reason for presentation, she stated, “I am not well,” and “I just want to be myself again.” In response to multiple questions, she took breaths as if to begin an answer but sighed heavily instead. During her initial interview, she also complained of a headache and of feeling “sad,” but she did not respond to clarifying questions seeking further information on the severity, character, duration, or other details regarding her symptoms. Intermittently, she pointed to various objects in the room, but the meaning of her gestures was not clear. Collateral information from family was sought but not obtained in the emergency department prior to admission. Chart review was performed (discussed below). The mental status exam was consistent with the above, notable for slowed speech, slowed and somewhat stereotyped movements, loose associations, poor attention, and disorientation. Laboratory testing revealed no gross abnormalities, with normal and negative results on complete blood count, comprehensive metabolic panel, thyroid testing, pregnancy screening, and urine toxicology (Table 1). A noncontrast CT of LW's head noted generalized volume loss much greater than expected for age. As she appeared to be medically stable with significant cognitive impairment of an unknown origin, she was admitted to inpatient psychiatry with a presumptive diagnosis of catatonia and to rule out a diagnosis of early-onset major neurocognitive disorder concerning volume loss on head CT.

On chart review and further collateral information obtained during her psychiatric admission, she appeared to have had limited contact with medical providers despite her significant impairment in functioning over the prior few years. Her most recent encounter had occurred nine months prior to this emergency room presentation, when she was evaluated by outpatient neurology for concerns for cognitive decline with unclear chronicity, depression, and reports of recent sexual assault by her father. At that time, her ex-mother-in-law provided collateral information, although she was unable to provide full information regarding LW's timeline of decline. On that visit, LW had endorsed difficulty with words and felt “dumb.” She was noted to have previously worked as a home health nurse eight years prior, at which time she was married, driving, and not noted to have any significant cognitive or verbal deficits. Furthermore, while she exhibited minor word-finding difficulty, she was noted to be mostly intact in expressive and receptive language abilities, which contrasted significantly with this emergency room presentation. Her neurologic exam was otherwise normal, with no deficits in the motor exam, reflexes, somatosensory exam, coordination, or gait. At that time, her decline and symptoms were attributed to depression and psychological trauma from her sexual assault, and she was referred to outpatient psychology. Unfortunately, she was lost to follow-up, and her next presentation was the emergency room visit described above.

Notable in this case was the difficulty that providers experienced in attaining collateral information or elaboration of the patient's history and symptoms. The primary source of collateral information was her ex-mother-in-law, whom LW had been living with for three years prior to presentation but had otherwise not contacted her since LW's divorce nearly a decade prior. Her ex-mother-in-law stated that LW was in contact with her for help three years prior to presentation and, at that time, was “child-like” in terms of her ability to care of herself or perform tasks. The ex-mother-in-law provided food, shelter, and support, but medical evaluation was limited to a primary care visit and the subsequent neurology appointment described above. She also revealed that LW's mother had died at a young age of an unknown illness. She adamantly denied knowledge of substance use by LW but emphasized that she had significant gaps in her knowledge of LW's activities and whereabouts prior to living with her. Possibly because of stigma related to perceived mental illness and family schism after sexual assault from LW's other family member, minimal further information was forthcoming despite repeated attempts via phone and in-person consultation.

As noted above, LW's presumptive diagnosis was catatonia, and because of her unknown family history and onset of symptoms reported in her late 20s or early 30s, neurodegeneration appeared less likely. Her reports of depression following severe trauma also appeared consistent with this diagnosis. Her symptoms suggestive of catatonia were evaluated with the Bush–Francis Catatonia Rating Scale as follows (note that domains are rated 0–3 in severity):

  Excitement: 0–absent
  Immobility: +2–virtually no interaction with the external world
  Mutism: +2–speaks <20 words per 5 min
  Staring: +2–gaze held >20 seconds with shifts in attention
  Posturing: +2–sitting without reacting 1–15 minutes at a time
  Grimacing: 0–absent
  Echopraxia/echolalia: +1–occasional mimicking of the examiner
  Stereotypy: +1–occasional repetitive, non-goal-oriented touching of the head with hand
  Mannerisms: 0–absent
  Verbigeration: +1–repeated phrases, such as “I don't know” and “I'm sorry”
  Rigidity: 0–absent
  Negativism: +1–mild, nonpurposeful resistance to attempts to move the patient during exams
  Waxy flexibility: 0–absent
  Withdrawal: +2–minimal food/fluid intake or interaction for >1 day
  Total: 14

Additional Bush–Francis domains were negative, including impulsivity, automatic obedience, mitgehen, gegenhalten, ambitendency, grasp reflex, perseveration, combativeness, and autonomic abnormalities.

**Figure 1:**
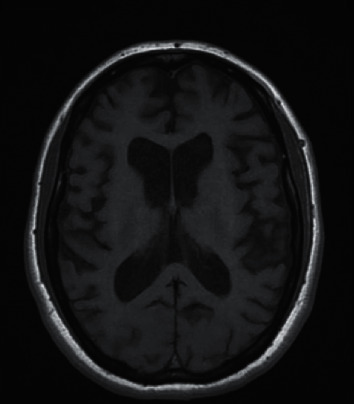
Axial T1-weighted MRI of the brain was performed on a 1.5 Tesla scanner showing generalized moderate cortical atrophy for the patient's age.

Unfortunately, LW did not recover with an initial treatment of catatonia, including a robust benzodiazepine trial (lorazepam 1 mg TID that was uptitrated to 2 mg TID). Neurology consult was placed, and it was noted that her symptoms appeared to improve with distraction, with LW showing the ability to briskly answer certain questions without difficulty. At this time, neurology service stated that her clinical syndrome and neurologic exam were most suggestive of primary psychiatric etiology. On initial consult, neurology service stated, “there is nothing in neither history nor exam to suggest primary neurologic process; however, MRI showing moderate volume loss in concerning (Figure 1). Suspect MRI findings are multifactorial. Considering possible degenerative process, although this would be highly unlikely at this young age, we would expect to see a specific pattern of atrophy on MRI.” Differential diagnosis was left broad, however, and EEG and further lab tests were pursued. EEG revealed diffuse slowing, which is a nonspecific finding of diffuse cortical dysfunction. Neuropsychological testing showed severe-to-profound impairment in all domains of cognition, with the patient scoring 20/144 on the Dementia Rating Scale.

Because of ongoing concerns for null or partial response to benzodiazepine treatment and incomplete explanation for cortical volume loss, lumbar puncture and further laboratory tests were conducted. These included broad panels for autoimmune, infective, and genetic causes of dementia (Table 1). ECT, a standard treatment for treatment-resistant catatonia, was considered but delayed until further diagnostic clarity could be reached because of family concerns regarding the procedure. Ultimately, genetic testing showed a heterozygous mutation for PSEN1 c.1292 C > A, a rare pathogenic mutation for autosomal dominant early-onset Alzheimer's disease (AD). Further history showed that her mother was from Jalisco, Mexico, known to be a hotspot for this mutation [1].

## 2. Discussion

We report a case of a 35-year-old woman with significant deterioration in psychomotor functioning, depression, and catatonic features, who was found to have the early-onset dementia-causing PSEN1 mutation. Early-onset AD occurs prior to 65 years of age [2, 3]. Pathologically, it is characterized by the accumulation of intracytoplasmic neurofibrillary tangles and amyloid deposition in the parenchyma and blood vessels [4]. Familial AD accounts for 0.5% of all cases [5]. Approximately 50% of familial AD cases have mutations in one of three genes: PSEN1, PSEN2, or APP [5]. PSEN1 is the most common of the three mutations and has multiple variants. The A431 E mutation in the PSEN1 gene is found in families whose roots originate in the state of Jalisco in Mexico.

Patients with PSEN1 tend to have the earliest age of onset, with a mean of 43.3 years [5] and a range of 30–55 years [2, 4]. PSEN1 is also most likely to present with atypical features, such as seizures, myoclonus, and cerebellar signs [5]. In a systematic review, 13.7% of patients with the PSEN1 mutation had depression, and 10.3% had personality changes [5]. In a case series of 128 individuals with a PSEN1 mutation, the most common initial clinical presentation was progressive memory loss with changes in behavior and personality [6]. Interestingly, like our patient, 75% of the sample had severe headaches prior to the onset of dementia [6].

Catatonia in the DSM-5 is defined as the presence of three or more of the following symptoms: cataplexy, waxy flexibility, stupor, agitation (not influenced by external stimuli), mutism, negativism, posturing, stereotypies, mannerisms, grimacing, and echolalia/echopraxia [7]. Benzodiazepines are the mainstay of treatment in catatonia. [8] Furthermore, response to benzodiazepines validates the diagnosis of catatonia [9]. Some clinicians use the lorazepam challenge test in which 1–2 mg of lorazepam is infused, and the patient is reevaluated for improvement [9]. ECT should be considered in catatonic patients not responding to medical treatment [9]. A recent review revealed that the response to ECT in catatonia ranged from 53% to 93%, and that the response was better in patients with mood disorders than in those with psychotic disorders [9].

Our patient's young age, sexual trauma history, apathy, anorexia, psychomotor slowing, and mutism are all symptoms expected in depression with catatonia. However, the lack of improvement with first-line medications (70%–80% of patients with catatonia are known to achieve full remission) and the presence of apraxia were more characteristic of neurodegenerative disorders [9]. In addition, global cortical atrophy might be more characteristic of dementia than depression. A recent meta-analysis examined subcortical brain alteration in depression and found that depressed patients have smaller hippocampal volumes than controls; however, this study did not show a significant difference in the total intracranial volume between patients with depression and controls [10]. Thus, diffuse intracranial volume loss in a depressed patient should prompt additional testing, especially if the patient is exhibiting treatment resistance.

Familial AD, although rare, should be considered in patients with progressive decline in activities of daily living when other more common etiologies have been excluded. Diagnosis in this case was challenging in the absence of family history and with reported trauma. This case is a cautionary reminder for clinicians that end stages of dementia can present similar to catatonia with mutism, lack of spontaneous movement, and refusal to eat. The clues to the diagnosis were profound generalized cortical atrophy in the MRI and lack of improvement with optimal medical management.

## Figures and Tables

**Table 1:** Laboratory tests performed.

Test	Result
Initial testing	
Complete metabolic panel and liver function tests	Within normal limits
Complete blood count	Within normal limits
Urine analysis	Within normal limits
Thyroid-stimulating hormone	0.97 (0.27–4.20) uIU/mL
Chest X-ray	Unremarkable
Urine toxicology	Amphetamines screen: negative
	Barbiturates screen: negative
	Cocaine screen: negative
	Benzodiazepine screen: negative
	THC screen: negative
	Methadone: negative
	Oxycodone screen: negative
	Opiates screen: negative
	Phencyclidine screen: negative
Secondary workup	
HIV 1/2 rapid antibody	Nonreactive
Rapid plasma reagin	Negative
Sedimentation rate	10 (0–20) mm/hr
C-reactive protein	<0.03 (<0.5) mg/dL
Autoimmune panel	Antinuclear antibody: negative
	Antineutrophil cytoplasmic Ab: negative
	C4, 29 (10–40) mg/dL
	C3, 139 (90–180) mg/dL
	SSA Ab <0.3 (0.0–6.9) U/mL
	SSB Ab <0.3 (0.0–6.9) U/mL
	Anti-DSDNA 7 (0–24) IU
	Smith antibody 1.5 (0.0–6.9) U/mL
	Anti-TPO: negative
	Mayo dementia, autoimmune evaluation panel: negative
Cerebrospinal fluid studies	
Color and clarity	Clear and colorless
White blood cells	1 (0–10) mm^3^
Red blood cell count	1 mm^3^
Protein	19 (15–45) mg/dL
Glucose	64 (40–70) mg/dL
Myelin basic protein	Negative
Oligoclonal bands	Negative
Cytology	No atypical lymphoid cells
Mayo paraneoplastic panel	Negative
Mayo dementia, autoimmune evaluation panel	Negative
Protein 14-3-3	<0.2
Neuronal specific enolase	Negative
VDRL	Negative

## Additional Points

Clinical pearl: end stages of dementia can present similarly to catatonia with mutism, lack of spontaneous movement, and refusal to eat.

## References

[1] Yescas P., Huertas-Vazquez A., Villarreal-Molina M. T. (2006). Founder effect for the Ala431Glu mutation of the presenilin 1 gene causing early-onset Alzheimer’s disease in Mexican families. *Neurogenetics*.

[2] Klimkowicz-Mrowiec A., Bodzioch M., Szczudlik A., Slowik A. (2014). Clinical presentation of early-onset alzheimer’s disease as a result of mutation in exon 12 of the PSEN-1 gene. *American Journal of Alzheimer’s Disease & Other Dementiasr*.

[3] Liu J., Wang Q., Jing D. (2019). Diagnostic approach of early-onset dementia with negative family history: implications from two cases of early-onset alzheimer’s disease with de Novo PSEN1 mutation. *Journal of Alzheimer’s Disease*.

[4] Hedera P., Turner R. S. (2002). Inherited dementias. *Neurologic Clinics*.

[5] Shea Y.-F., Chu L.-W., Chan A. O.-K., Ha J., Li Y., Song Y.-Q. (2016). A systematic review of familial Alzheimer’s disease: differences in presentation of clinical features among three mutated genes and potential ethnic differences. *Journal of the Formosan Medical Association*.

[6] Lopera F., Ardilla A., Martinez A. (1997). Clinical features of early-onset Alzheimer disease in a large kindred with an E280A presenilin-1 mutation. *JAMA: The Journal of the American Medical Association*.

[7] Tandon R., Heckers S., Bustillo J. (2013). Catatonia in DSM-5. *Schizophrenia Research*.

[8] Pelzer A., van der Heijden F., den Boer E. (2018). Systematic review of catatonia treatment. *Neuropsychiatric Disease and Treatment*.

[9] Sienaert P., Dhossche D. M., Vancampfort D., De Hert M., Gazdag G. (2014). A clinical review of the treatment of catatonia. *Frontiers in Psychiatry*.

[10] Schmaal L., Veltman D. J., Veltman D. J. (2016). Subcortical brain alterations in major depressive disorder: findings from the ENIGMA Major Depressive Disorder working group. *Molecular Psychiatry*.

